# Contribution of Dopamine D1/5 Receptor Modulation of Post-Spike/Burst Afterhyperpolarization to Enhance Neuronal Excitability of Layer V Pyramidal Neurons in Prepubertal Rat Prefrontal Cortex

**DOI:** 10.1371/journal.pone.0071880

**Published:** 2013-08-20

**Authors:** Feng Yi, Xue-Han Zhang, Charles R. Yang, Bao-ming Li

**Affiliations:** 1 Institute of Neurobiology and State Key Laboratory of Medical Neurobiology, Institutes of Brain Science, Fudan University, Shanghai, China; 2 CNS Pharmacology and Ion Channel, Shanghai Chempartner Co. Ltd., Shanghai, China; 3 Center for Neuropsychiatric Diseases, Institute of Life Science, Nanchang University, Nanchang, China; Goethe University Frankfurt, Germany

## Abstract

Dopamine (DA) receptors in the prefrontal cortex (PFC) modulate both synaptic and intrinsic plasticity that may contribute to cognitive processing. However, the ionic basis underlying DA actions to enhance neuronal plasticity in PFC remains ill-defined. Using whole-cell patch-clamp recordings in layer V-VI pyramidal cells in prepubertal rat PFC, we showed that DA, via activation of D1/5, but not D2/3/4, receptors suppress a Ca^2+^-dependent, apamin-sensitive K^+^ channel that mediates post-spike/burst afterhyperpolarization (AHP) to enhance neuronal excitability of PFC neurons. This inhibition is not dependent on HCN channels. The D1/5 receptor activation also enhanced an afterdepolarizing potential (ADP) that follows the AHP. Additional single-spike analyses revealed that DA or D1/5 receptor activation suppressed the apamin-sensitive post-spike mAHP, further contributing to the increase in evoked spike firing to enhance the neuronal excitability. Taken together, the D1/5 receptor modulates intrinsic mechanisms that amplify a long depolarizing input to sustain spike firing outputs in pyramidal PFC neurons.

## Introduction

The prefrontal cortex (PFC) is functionally engaged in organizing and processing cognitive information that are critical to the planning and execution of forthcoming behaviors [1]. Neural activities in the PFC that represent working memory processing can be modulated by mesocortical DA input, predominantly via activation of D1/5 receptors [2], [3].

At the cellular levels, DA is known to modulate not only synaptic, but also intrinsic plasticity in the PFC to generate repetitive and persistent firing [4]–[6]. Intrinsic plasticity is integral to synaptic plasticity, and occurs through modulation of postsynaptic soma-dendritic ion channels that regulate neuronal excitability, thus allowing coupling of potentiated excitatory postsynaptic potentials (EPSPs) (from synaptic inputs) to reach spike firing (output) [7]. The resultant prolonged changes in spike firing are now termed long-term potentiation (or depression) of neuronal excitability (LTP/D-IE) [7].

During sustained repetitive firing of a PFC neuron, the firing of single spike or bursts of spikes is temporarily terminated by a post-spike/burst afterhyperpolarization (AHP) that provides varying inter-spike or inter-burst inactive intervals. AHP is both intracellular and extracellular Ca^2+^-dependent [8]–[10] and consists of three subtypes: 1) fast AHP, (mediated by BK channel) [11], 2) apamin-sensitive medium AHP (mediated by a Ca^2+^-dependent K^+^ ion conducting SK channel), and 3) slow AHP (mediated by an as yet unknown channel [12]. Recent pharmacological and knockout mice data suggest that both SK and Kv7/KCNQ/M channels can also mediate cortical mAHP [13], [14].

Modulation of the AHP will alter neuronal refractoriness, inter-spike intervals, and consequently, spike timing, firing patterns and frequencies, which ultimately lead to changes in neuronal excitability [7], [15], [16]. An increase in post-burst slow AHP in hippocampal CA1 neurons is found in aged, cognitively-impaired, but not cognitively-unimpaired rodents. In contrast, in prepubertal animals, a predominant presence of the smaller AHP facilitates neuronal excitability increase following different forms of associative learning 17,18, presumably the smaller AHP is being modulated by learning-related release of neuromodulators, including dopamine.

Membrane hyperpolarization during AHP can, in turn, activate an h-current (I_h_) that is mediated by the hyperpolarization-activated and cyclic nucleotide-gated cationic HCN1 and/or HCN2 channels. Activated I_h_ slowly depolarizes the cell back towards its equilibrium potential (V_R_ = −30 mV). I_h_ is functionally important for regulating neuronal excitability, resting potential and rhythmic pacemaker activity [19]–[21]. Thus, AHP and I_h_ interact to shape the amplitude and duration of post-spike/burst AHP, as shown in hippocampal CA1, thalamic relay neurons and striatal cholinergic interneurons [22]–[24].

DA, through D1/5 receptor activation, enhances neuronal excitability of PFC neurons in pre-pubertal PFC neurons in vitro and mature PFC neurons in vivo (see Table 1 for a summary of the literature for this DA effects on PFC neuronal excitability). This suggests that the pre-pubertal PFC is already endowed with the functional D1/5 receptors [25] that can modulate intrinsic mechanisms that contributed to neuronal excitability enhancement. This DA increase in neuronal excitability may promote local interconnected network interaction and forms part of the mechanisms for a sustained increase in persistent firing necessary for cognitive processes [2], [5].

**Table 1:** A summary of published findings of DA-induced changes in neuronal excitability recorded from rat PFC neurons of different postnatal ages [4], [26], [27], [29], [30], [41]–[43], [78]–[89].

Published Studies	Age	Animal	Neuronal types	Treatment	Effect on single neuronal excitability
Bandyopadhyay S et al., 2005	25–30 d	Rat	PFC pyramidal cell layer II/III	SKF81297	increase
Ceci A et al., 1999	150–250 g	Rat	PFC pyramidal cell	DA	increase
Chen et al., 2007	27–36 d	Rat	PFC layer V/VI pyramidal cell	SKF81297	increase
Geijo-Barrientos E et al., 1995	150–200 g	Rat	PFC pyramidal cell	DA	decrease
Gorelova & Yang, 2000	35–49 d	Rat	PFC layer V/VI pyramidal cell	SKF81297	increase
Gulledge AT & Jaffe DB., 2001	17–30 d	Rat	PFC layer V pyramidal cell	DA	increase
Gulledge AT et al., 1998	17–30 d	Rat	PFC layer V pyramidal cell	DA	decrease
Henze DA et al., 2000	Young	Monkey	PFC layer V pyramidal cell	DA	increase
Lavin A et al., 2005	250–300 g	Rat	PFC deep layer pyramidal cell	VTA stimulation	increase
Lavin, A & Grace, AA, 2001	14–21 d	Rat	PFC deep layer pyramidal cell	SKF38393	increase
Moore A et al., 2011	19–32 d	Rat	PFC layer V pyramidal cell	DA	increase
Di Pietro & Seamans 2011	21–35 d	Rat	PFC pyramidal cell	DA	increase
Penit-Soria J et., 1987	18–23 d	Rat	PFC layer III/V/VI pyramidal cell	DA	increase
Rotaru DC et al., 2007	19–28 d	Rat	PFC layer V pyramidal cell	SKF81297	decrease
Seamans JK et al., 2001	14–28 d	Rat	PFC layer V interneuron	DA	bidireactional
Seong & Carter, 2012	21–28 d	Mice	PFC layer V D1+ve pyramidal cell	SKF38393	increase
Thurley et al., 2008	17–44 d	Rat	PFC layer V pyramidal cell	SKF38393	increase
Tseng & O’Donnell, 2004	42–65 d	Rat	PFC layer V/VI pyramidal cell	SKF81297	increase
Wang & O’Donnell, 2001	24–28 d	Rat	PFC layer V pyramidal cell	SKF38393	increase
Yang CR, Seamans JK, 1996	80–100 g	Rat	PFC layer V/VI pyramidal cell	SKF81297	increase

The DA enhancement of neuronal excitability depends on an Ca^2+^-dependent intracellular signaling [4], [26], [27], which may in turn modulate the i[Ca^2+^]-dependent post-spike/burst AHP. In the present study, we characterized the modulatory effects of DA on post-spike/burst AHP in layer V/VI pyramidal cells of prepubertal rat PFC, and the contribution of modulated AHP to the overall neuronal excitability changes. We found that the D1/5 receptor suppressed post-spike/burst mAHP and enhanced a post-AHP afterdepolarizing potential (ADP), all independent of I_h_ activation, to contribute to the increase in neuronal excitability of layer V-VI PFC neurons in prepubertal rats. The findings also enable us to understand DA actions in the still developing PFC.

## Materials and Methods

### Ethics Statement

The present study was strictly in compliance with Guide for the Care and Use of Laboratory Animals of the National Institutes of Health. All the experimental protocols were approved by the Committee on the Ethics of Animal Experiments of the Fudan University (Permit Number: 2007-0002). All surgery was performed under deeply anesthesia with sodium pentobarbital, and all efforts were made to minimize suffering.

### Brain Slice Preparation

Male Sprague-Dawley (18–25 d, Chinese Academy of Sciences, Shanghai, China) rats, housed in normal (12∶12) hr light-dark cycle (temperature 25°C, humidity 50%), were anesthetized with sodium pentobarbital (40 m g/kg, intraperitoneal). After decapitation, brains were immediately placed in ice-cold artificial cerebrospinal fluid (ACSF) bubbled with 95% O_2_-5% CO_2_, and containing (in mM): 124 NaCl, 26 NaHCO_3_, 3 KCl, 0.5 CaCl_2_, 4 MgSO_4_, 0.4 ascorbic acid and 10 glucose (pH 7.4). Bilateral coronal slices (400-µm-thick) containing the medial PFC were cut using a vibratome (1000+, Pelco 102, Tedpella, USA). The cut brain slices were placed immediately in warm (35°C) continuously oxygenated ACSF, containing (in mM) 124 NaCl, 26 NaHCO_3_, 3 KCl, 2.3 CaCl_2_, 1.3 MgCl_2_, and 10 glucose. After 30 min, the slices were cooled to room temperature (25°C) in the same ACSF for the rest of the day.

### Whole-cell Patch Clamp Recordings

After at least 1 hour of incubation at room temperature, a single slice was transferred to the recording chamber on an upright optical microscope (T2, Olympus, Japan). The perfusion ACSF during slice recording (at room temperature, maintained at ∼ 25°C by air-conditioning) was delivered with a peristaltic pump (Peri-Star 291, World Precision Instruments, Sarasota, FL) at a rate of 2–3 ml/min. Whole-cell patch-clamp recordings were made under DIC-IR video microscopy (QIMAGE FAST 1394, Ddisoftware Instruments, USA). Patch pipettes were prepared from borosilicate glass capillaries (G120TF-3; Warner Instruments, USA) using a micropipette puller (P97; Sutter Instruments, Novato, CA, USA). Pipettes (3–5 MΩ) were filled with internal solution containing (in mM) 150 K^+^ gluconate, 0.4 EGTA, 8 NaCl, 2 ATP. Mg, 0.1 GTP.Na^+^_3_, 10 HEPES, and 10 Na_2_phosphocreatine with pH adjusted to 7.2–7.4 by KOH, and had an osmolarity of 280–295 mOsm.

Whole-cell voltage signals in current-clamp modes were collected with an AxoPatch 200B amplifier (Molecular Devices, Sunnyvale, CA., USA) in layer V/VI pyramidal cells in medial PFC. Pyramidal cells were identified by their morphological and electrophysiological features. Layer V/VI pyramidal cell with a prominent apical dendrite has the typical electrophysiological characteristic of spike frequency adaptation upon injection of a depolarizing pulse (800 msec).

APV (50 µM), DNQX (20 µM) and Picrotoxin (100 µM) were bath-applied *throughout* the experiment to block excitatory (glutamate) and inhibitory (GABAa) inputs to the recorded cells, so that the recorded cells were synaptically isolated from other cells.

Whole-cell recordings in current-clamp mode were used to test the neuronal excitability and post-burst/spike AHP of the mPFC neurons. The membrane potential was held at −55 mV manually by DC injection throughout the entire experiment.

The neuronal excitability test of the mPFC pyramidal cells was obtained by performing a spike activation protocol. The spike activation protocol contained a 200-ms and 30 pA hyperpolarizing pre-pulse (to monitor changes in the input resistance of recorded cells) and a 500-msecs depolarizing pulse that was adjusted to evoke 3–4 spikes in baseline recordings. The hyperpolarizing and depolarizing pulses were separated by 1 second. The post-burst AHP test consisted of a 13 pulse-train (50 Hz, 2 msec pulse-width, 2.8 nA). The excitability test and the post-spike/burst AHP recording were performed sequentially every 30 secs.

### Data Acquisition and Analyses

The biophysical data were digitized and recorded on-line using Clampex (version 9.2.0.09; Axon Instruments, Molecular Devices). The data analyses were performed using Clampfit (version 9.2.0.09; Axon Instruments, Molecular Devices). Cursors-defined mean amplitudes, area and decay time of AHP from 5 consecutive responses before drug application and before wash were taken for paired comparison. Significant main effects were evaluated by paired *t*-test or unpaired *t*-test or one-way ANOVA using Origin 8.1 (OriginLab, Northampton, MA) or SPSS Statistic 21 (SPSS, Chicago, IL). P<0.05 was deemed as significant difference. All data are reported as the means ± SEM.

The peak amplitude and integrated area of the post-burst AHP are measured when the neurons were held at −55 mV. The decay time constant of the post-burst AHP is defined as the time span from 10% to 90% of the amplitude, when the AHP is decaying back to the holding potential of −55 mV. For the ADP measurements, because the variation of the ADP time courses in the recorded cells was large, we fixed a time window (the third second after the beginning of each sweep of recording) to measure the amplitude and area of ADP at that time window after post-burst AHP.

For the post-single spike AHP analyses, there are three potential components that are analyzed in the present work. We name the three components as fAHP, fADP and mAHP. The amplitudes of the fAHP, fADP and mAHP are measured at their peak when the cell was held at −55 mV.

All measurements were made >10 min after membrane rupture to allow for adequate internal solution equilibration within the neuron. All traces of potentials shown in figures are average of 5 consecutive responses.

### Drug Application

Apamin, ATP.Mg, Dopamine hydrochloride (DA), GTP.Na^+^_3_, HEPES, K^+^ gluconate, Picrotoxin (PTX), Quinpirole and SKF81297 were purchased from Sigma Chemical Company (Sigma, St Louis, MO). ZD7288 were purchased from Tocris Cookson Ltd (Ellisville, MO). Other reagents in analytical reagent (AR) grade were purchased from the Shanghai Chemical Company, Shanghai, China.

Stock solutions of PTX (50 mM) was prepared in DMSO and stored at 4°C. Stock solutions of DNQX (20 mM) was also prepared in DMSO and stored at −20°C. Stock solutions of APV (25 mM) and quinpirole (10 mM) were prepared in ultrapure deionized water (Millipore Q-Gard 1, Billerica, MA) and stored at −20°C. SKF81297 was prepared as 2.5 mM stock in ascorbic acid (also 2.5 mM in stock) in the lab with lights turned off, and the stock solution was stored at −20°C for no longer than one month. When DA and SKF81297 were bath administered, the microscope light was also dimmed down to prevent photo degradation of the compounds.

## Results

**Figure 1:**
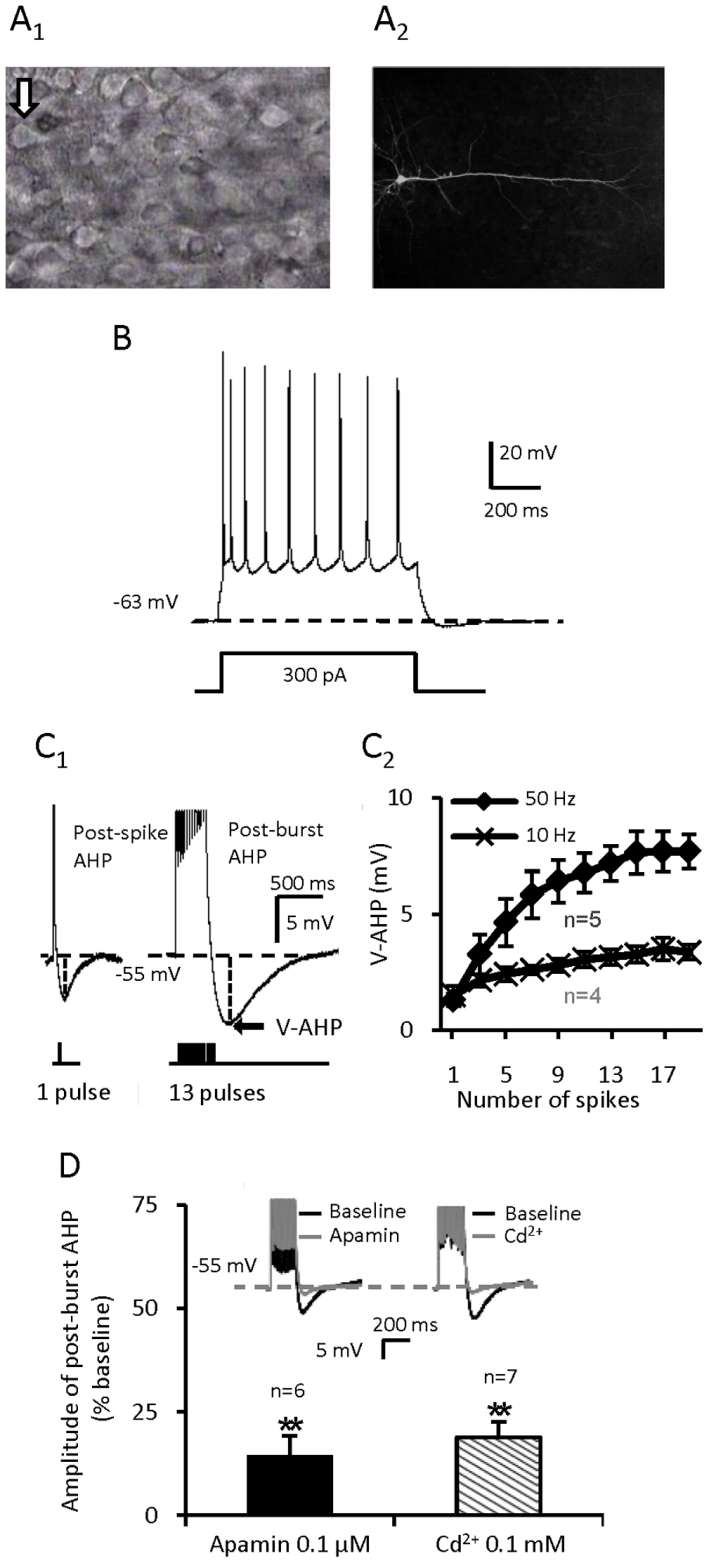
Identification of mPFC pyramidal cells and features of post-spike/burst AHP. (**A**) A pyramidal cell in layer V/VI of the mPFC with a prominent apical dendrite. (**B**) A mPFC pyramidal cell which shows spike-frequency adaptation. (**C**) C_1_: The post-spike AHP and post-burst AHP with 13 spikes. Each inward depolarizing pulse is 2.8 nA for 2 ms. C_2_: The more the no. of depolarizing pulses are and the higher the depolarizing frequency is, the bigger the amplitude of post-burst AHP is. (**D**) The induced post-burst AHP is apamin sensitive and calcium dependent (**p<0.01 for Apamin vs. baseline; **p<0.01 for Cd^2+^ vs. baseline, paired *t-*test). Values are represented as mean ± SE.

Whole-cell recordings were performed in rat layer V-VI mPFC pyramidal cells identified by their morphological and electrophysiological features. As shown in Fig. 1A and 1B, the recorded pyramidal cell had a prominent apical dendrite and showed typical spike frequency adaptation upon injection of 800 ms depolarizing pulse. All cells included in the database of the present study had resting potentials more negative than −60 mV and evoked spikes with amplitude greater than +70 mV.

For recordings of AHP, the basal membrane potential was held at −55 mV manually by DC injection. Post-spike AHP was elicited by a single short pulse (2 ms, +2.8 nA) (Fig. 1C). In our pilot studies, to optimize the recordings of the spike number-dependent post-burst AHP we found that a train of 13 pulses evoked at 50 Hz (2 ms per pulse, +2.8 nA), elicited post-burst AHPs with consistent amplitude and integrated area (Fig. 1C). To prevent synaptic feedback influences on the spike evoked AHP, all recordings were made in the presence of glutamate and GABA receptor antagonists.

As shown in Fig. 1D, the post-burst AHP was almost totally suppressed by bath-application of apamin (0.1 µM) (Amplitude: 14.31±4.93%, p<0.01, paired *t* test, n = 6; control was normalized to 100%) or Cd^2+^ (0.1 mM) (Amplitude: 18.87±3.82%, p<0.01, paired *t* test, n = 7), suggesting that the post-burst AHP recorded was mainly composed of the Ca^2+^- and apamin-sensitive medium AHP (mAHP) [14], [28]. With an increase in number of pulses and depolarizing pulses frequency, there was greater Ca^2+^ influx to elicit a greater V-AHP (the absolute AHP amplitude value that surpasses the holding potential, Fig. 1C).

### DA Enhances Neuronal Excitability and Suppresses Post-burst AHP

**Figure 2:**
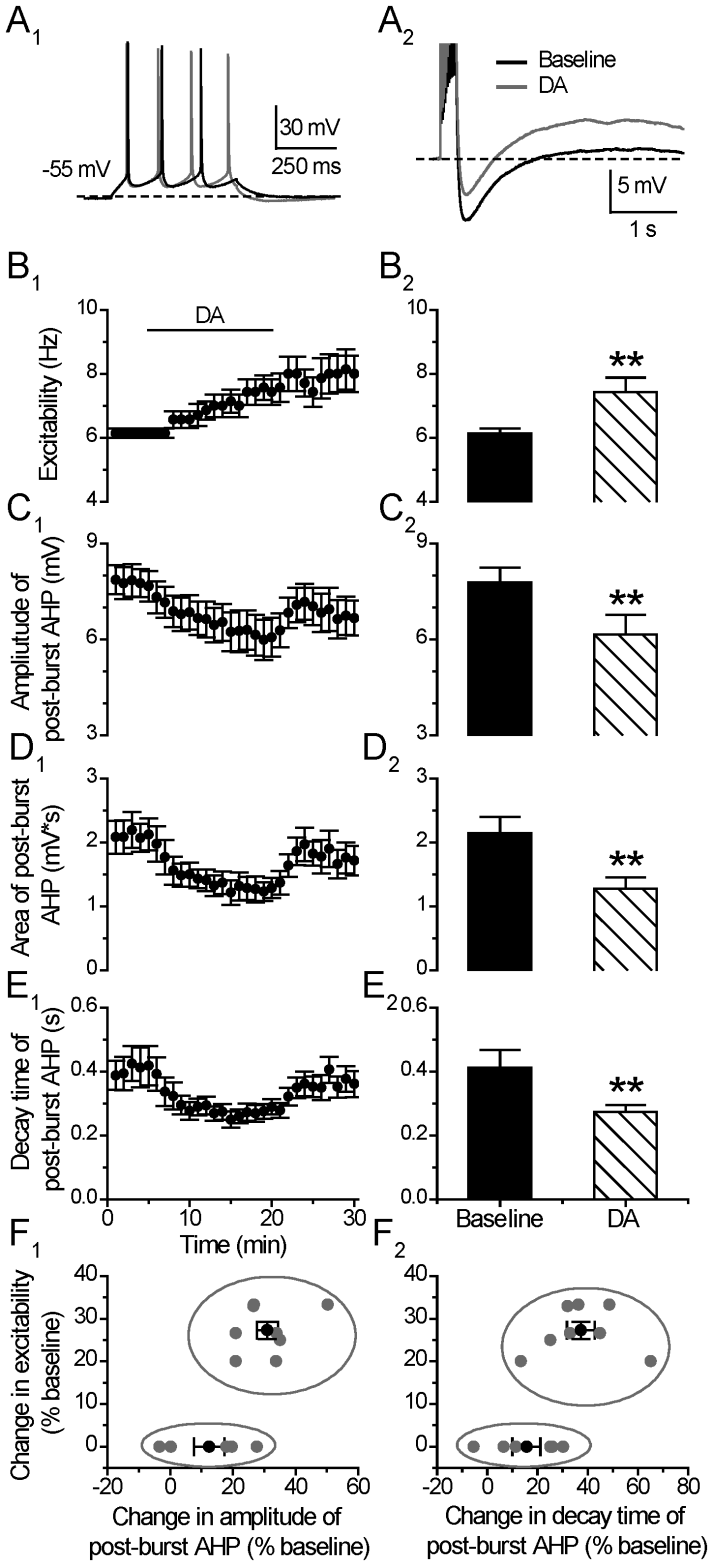
DA enhances neuronal neuronal excitability and suppresses post-burst AHP. (**A**) An example of the effect of DA (30 µM) on neuronal excitability (left) and post-burst AHP (right). The protocols for excitability and post-burst AHP recording were run alternately every 30 seconds. (**B**) Excitability before, during and after 15-min application of the DA (left). Group histograms showing that DA enhances neuronal excitability of mPFC neurons (right) (**p<0.01 for DA vs. baseline, paired *t-*test). (**C**) Amplitude of post-burst AHP before, during and after 15-min application of the DA (left). Group histograms showing that DA suppresses the amplitude of post-burst AHP (right) (**p<0.01 for DA vs. baseline, paired *t-*test). (**D**) Area of post-burst AHP before, during and after 15-min application of the DA (left). Group histograms showing that DA reduces the area of post-burst AHP (right) (**p<0.01 for DA vs. baseline, paired *t-*test). (**E**) Decay time of post-burst AHP before, during and after a 15-min application of the DA (left). Group histograms showing that DA reduces the decay time of post-burst AHP (right) (**p<0.01 for DA vs. baseline, paired *t-*test). *an increase in post-burst AHP.* (**F**) Correlation plot showing two potential clusters of neurons with distinct response of excitability to the application of DA. Mean±SEM of the two groups were displayed as black symbols. *In a few cells, the negative values in the X-axis mean an increase in post-burst AHP.*

The effects of DA on neuronal excitability and post-burst AHP were tested alternately in the same individual cells (Fig. 2A). After establishing a stable baseline recording for at least 5 minutes, DA (30 µM) was bath-applied for 15 minutes. As shown in Fig. 2B, there was an overall significant enhancement of the depolarization pulse-evoked spike number upon DA application (Excitability: from 6.14±0.15 to 7.37±0.37 Hz, p<0.01, paired *t* test, n = 14) and the evoked spike responses continued to increase with time, with the peak increase much after the termination of DA application (peak response in excitability: 8.00±0.59 Hz**,** p<0.01, paired *t* test, n = 14) (Fig. 2B1) [4].

It is notable that with the analysis window placed at the last 5 min of the DA application, 8 of the 14 tested cells (57%) were considered to have an enhanced excitability during the DA treatment, and the remaining 6 cells showed no obvious response *during* the DA application. However, 3 of these 6 cells showed enhanced excitability after the termination of DA application. Thus, 79% (11/14 cells) of the tested neurons showed an enhanced excitability by DA after the termination of DA application. The post-burst AHP during the last 5 min of the DA application was suppressed by DA application (AHP Amplitude: from 7.78±0.47 to 6.15±0.62 mV, p<0.01; Area: from 2.14±0.26 to 1.28±0.18 mV*s, p<0.01; Decay time constant: from 0.41±0.05 to 0.27±0.02 s, p<0.01, paired *t* test, n = 14) (Fig. 2C, 2D and 2E).

We found that the time course of the post-burst AHP change was quite different from that of the excitability change following DA application. In 12/14 tested neurons, the reduced post-burst AHP recovered to baseline level within a few minutes after the termination of DA application, whereas the post-DA enhanced excitability persisted well after termination of DA application and showed only a partial recovery during the washout.

To determine which neurons show this correlated responses of DA-induced reduction of AHP amplitude and increase in neuronal excitability, we plotted the % change of neuronal excitability (during the last 5 minutes of of DA application) against the % change of post-burst AHP for all the 14 tested neurons. As shown in Fig. 2F, the cells (n = 8) showing increased excitability to DA application also showed considerable suppression in post-burst AHP amplitude (31.04±3.39%). The other cells (n = 6) showing no increase in excitability during the last 5 mins of DA application showed smaller suppression of post-burst AHP amplitude (12.43±4.87% vs. 31.04±3.39%, p<0.01, one-way ANOVA). The decay time change in the two groups of cells are also significant different in responding to DA application (37.26±5.57% vs. 15.61±5.64%, p<0.05, one way ANOVA). These results suggest that there might be two groups of PFC neurons exhibiting different AHP responses during DA application to contribute to the neuronal excitability enhancement. Furthermore, it is also likely that DA enhanced neuronal excitability is biphasic, ie., during DA exposure, DA enhanced neuronal excitability through suppression of AHP, and during post-DA application, the excitability increase is maintained and reached peak responses slowly via other mechanisms not related directly to AHP changes.

### D1/5-receptor Stimulation Enhances Neuronal Excitability and Suppresses Post-burst AHP

**Figure 3:**
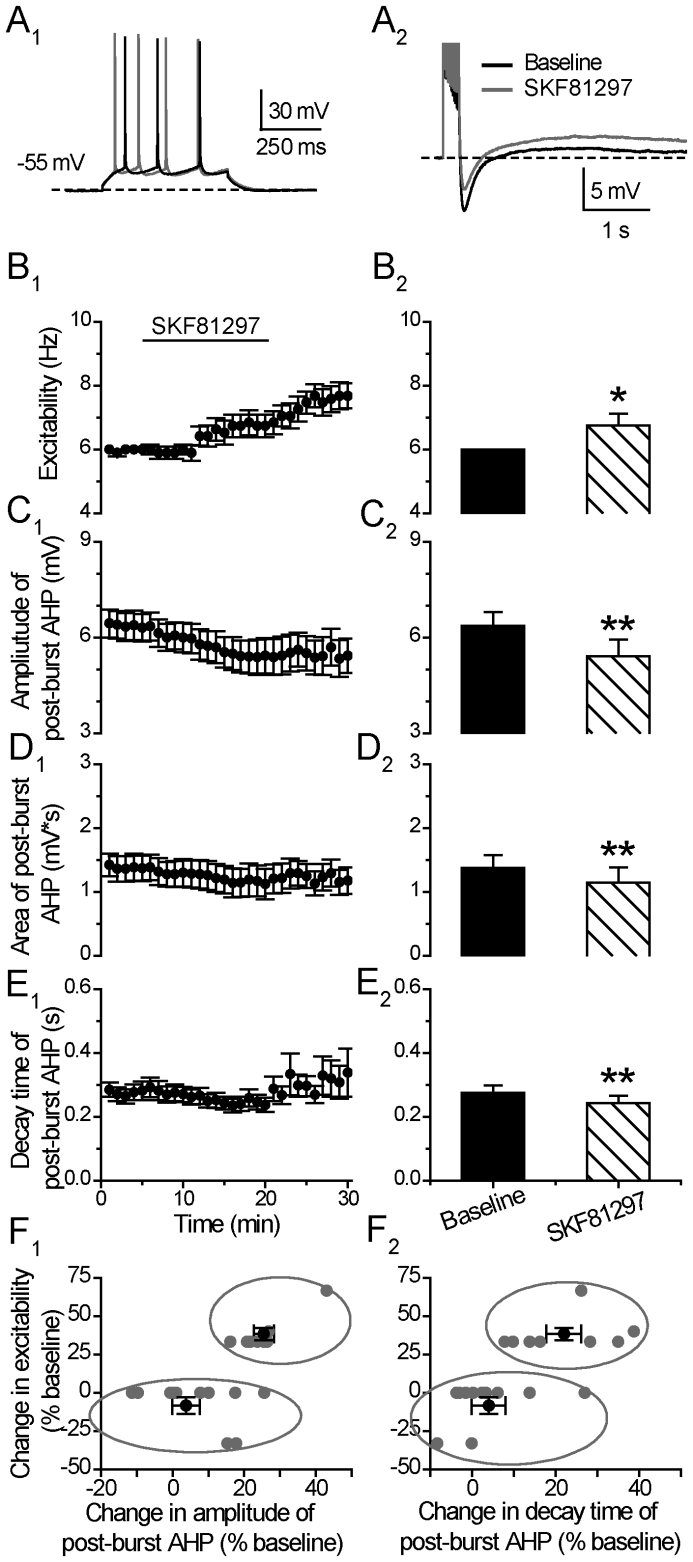
D1/5 stimulation enhances neuronal excitability and suppresses post-burst AHP. (**A**) An example of the effect of D1/5 receptor agonist SKF81297 (10 µM) on neuronal excitability (left) and post-burst AHP (right). The protocols for excitability test and post-burst AHP recording were run alternately every 30 seconds. (**B**) Excitability before, during and after 15-min application of the D1/5 receptor agonist SKF81297 (left). Group histograms showing that SKF81297 enhances the neuronal excitability of mPFC neurons (right) (*p<0.05 for SKF81297 vs. baseline, paired *t-*test). (**C**) Amplitude of post-burst AHP before, during and after 15-min application of SKF81297 (left). Group histograms showing that SKF81297 reduces the amplitude of post-burst AHP (right) (**p<0.01 for SKF81297 vs. baseline, paired *t-*test). (**D**) Area of post-burst AHP before, during and after 15-min application of SKF81297 (left). Group histograms showing that SKF81297 reduces the area of post-burst AHP (right) (**p<0.01 for SKF81297 vs. baseline, paired *t-*test). (**E**) Decay time of post-burst AHP before, during and after a 15-min application of SKF81297 (left). Group histograms showing that SKF81297 reduces the decay time of post-burst AHP (right) (**p<0.01 for SKF81297 vs. baseline, paired *t-*test). (**F**) Correlation plot showing two potential clusters of neurons with distinct response of excitability to the application of SKF81297. Mean±SEM of the two groups were displayed as black symbols. *In a few cells, the negative values in the X-axis mean an increase in post-burst AHP.*

To identify which DA receptor is likely to regulate this AHP mechanism at this time window, we first examined the effects of the D1/5 receptor agonist SKF81297 on neuronal excitability and post-burst AHP (Fig. 3A). Consistent with previous studies [29], stimulation of D1/5 receptors with SKF81297 (10 µM) significantly enhanced the neuronal excitability (Excitability: from 6.00±0.00 to 6.76±0.36 Hz, p<0.05, paired *t* test, n = 19) (Fig. 3B). Towards the end of the 15 min agonist application, 8 out of the 19 cells (42%) showed an increased spike numbers. In the remaining 11 cells, 5 cells showed enhanced excitability only after the termination of D1/5 agonist. The other 6 cells (32%) showed no obvious response to SKF81297 throughout the recordings. Thus, in a total of 68% (13 of 19 cells) of the tested cells, the enhanced excitability persisted and peaked after the termination of SKF81297 application (Excitability: from 6.00±0.00 to 7.62±0.38 Hz, p<0.05, paired *t* test, n = 19). Among the 19 tested cells, a large part of the cells exhibited a suppressed post-burst AHP *during* the application of SKF81297 (Amplitude: from 6.37±0.43 to 5.42±0.52 mV, p<0.01; Area: 1.38±0.19 to 1.15±0.23 mV*s, p<0.01; Decay time constant: from 0.28±0.02 to 0.24±0.02 s, p<0.01; paired *t* test, n = 19) (Fig. 3C, 3D and 3E). Notably, following application of the agonist SKF81297, the suppressed AHP amplitude persisted longer than that after DA application and showed little recovery during the wash period (compare Fig. 2C_1_ with Fig. 3C_1_).

**Figure 4:**
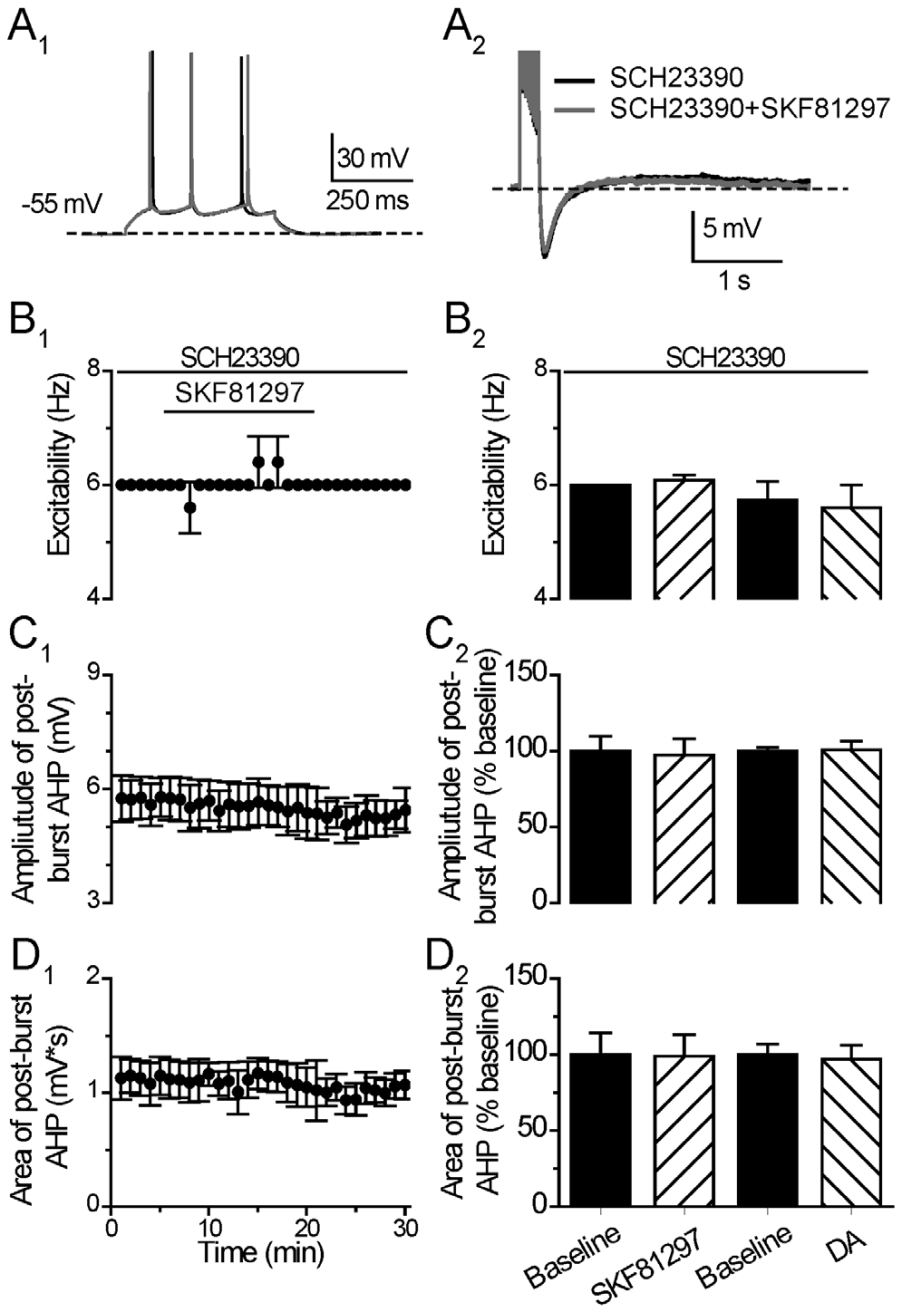
D1/5R antagonist blocks the effect of D1/5R or DA on neuronal excitability and post-burst AHP. (**A**) An example of the effect of D1/5 receptor agonist SKF81297 (10 µM) on neuronal excitability (left) and post-burst AHP (right) in the presence of D1/5 receptor antagonist SCH23390 (10 µM). The protocols for excitability test and post-burst AHP recording were run alternately every 30 seconds. (**B**) Excitability before, during and after 15-min application of SKF81297 in the presence of SCH23390 (left). Group histograms showing that SKF81297 or DA does not suppress the neuronal excitability of mPFC neurons in the presence of SCH23390 (right) (p>0.05 for SKF81297+SCH23390 vs. baseline; p>0.05 for DA+ SCH23390 vs. baseline, paired *t-*test). (**C**) Amplitude of post-burst AHP before, during and after 15-min application of SKF81297 in the presence of SCH23390 (left). Group histograms showing that SKF81297 or DA does not suppress the amplitude of post-burst AHP in the presence of SCH23390 (right) (p>0.05 for SKF81297+ SCH23390 vs. baseline; p>0.05 for DA+ SCH23390 vs. baseline, paired *t-*test). (**D**) Area of post-burst AHP before, during and after 15-min application of the SKF81297 in the presence of SCH23390 (left). Group histograms showing that SKF81297 or DA does not suppress the area of post-burst AHP in the presence of SCH23390 (right) (p>0.05 for SKF81297+ SCH23390 vs. baseline; p>0.05 for DA+ SCH23390 vs. baseline, paired *t-*test).

The D1/5 agonist-induced enhancement of excitability and suppression on post-burst AHP was blocked in the presence of 10 µM SCH23390 before the termination of SKF81927 application (Excitability: from 6.00±0.00 to 6.08±0.08 Hz, p = 0.37; Post-burst AHP amplitude: from 7.44±0.70 to 7.22±0.82 mV, p = 0.11; Area: from 1.46±0.2 to 1.25±0.2 mV*s, p = 0.68, paired *t* test, n = 5) (Fig. 4B, 4C and 4D). Furthermore, the DA effects on excitability and post-burst AHP were also blocked by the co-administered SCH23390 before the termination of DA application (Excitability: 5.73±0.32 to 5.60±0.4 Hz, p = 0.17; Post-burst AHP Amplitude: from 10.00±1.43 to 10.01±1.55, p = 0.89; Area: 6.73±2.15 to 6.63±1.98 mV*s p = 0.73, paired *t* test, n = 6) (Fig. 4B, 4C and 4D). Notably, in the absence of SCH23390, a large number of cells showed enhanced excitability after the termination of SKF81297 (13/19) or DA (11/14), while, in the presence of SCH23390, no cell showed obvious change in excitability during the same post-drug time period after SKF81297 (n = 5) or DA (n = 6) application, suggesting that the D1/5 agonist or DA influences on neuronal excitability and post-burst AHP were mediated through the D1/5 receptors.

In the correlation plots in Fig. 3F1 and 3F2, we found that the cells (n = 8) showing increased excitability to SKF81297 application also showed considerable suppression in post-burst AHP amplitude (25.47±2.79%), while the other cells (n = 11) showed a smaller suppression of post-burst AHP amplitude (6.70±3.57% vs. 25.47±2.79%, p<0.01, one-way ANOVA). The decay time changes in the two parts of cells are also significantly different in responding to SKF81297 application (3.25±2.94% vs. 21.95±4.13%, p<0.01, one-way ANOVA).

Seong and Carter [30] recently reported that D1 receptors are present in a portion of layer V pyramidal cells in the PFC. We found that cells that respond to DA and D1/5-receptor agonist in excitability typically have a higher input resistance (198.86±14.79 vs. 155.72±10.15 MOhm, p<0.05, unpaired t test) and smaller I_h_ tendency (Sag %, 17.88+10.81% vs. 28.22±13.05%, p = 0.052, unpaired t test), which are similar with the findings reported [30].

### D2/3/4-receptor Stimulation Produces No Effect on Neuronal Excitability and Post-burst AHP

**Figure 5:**
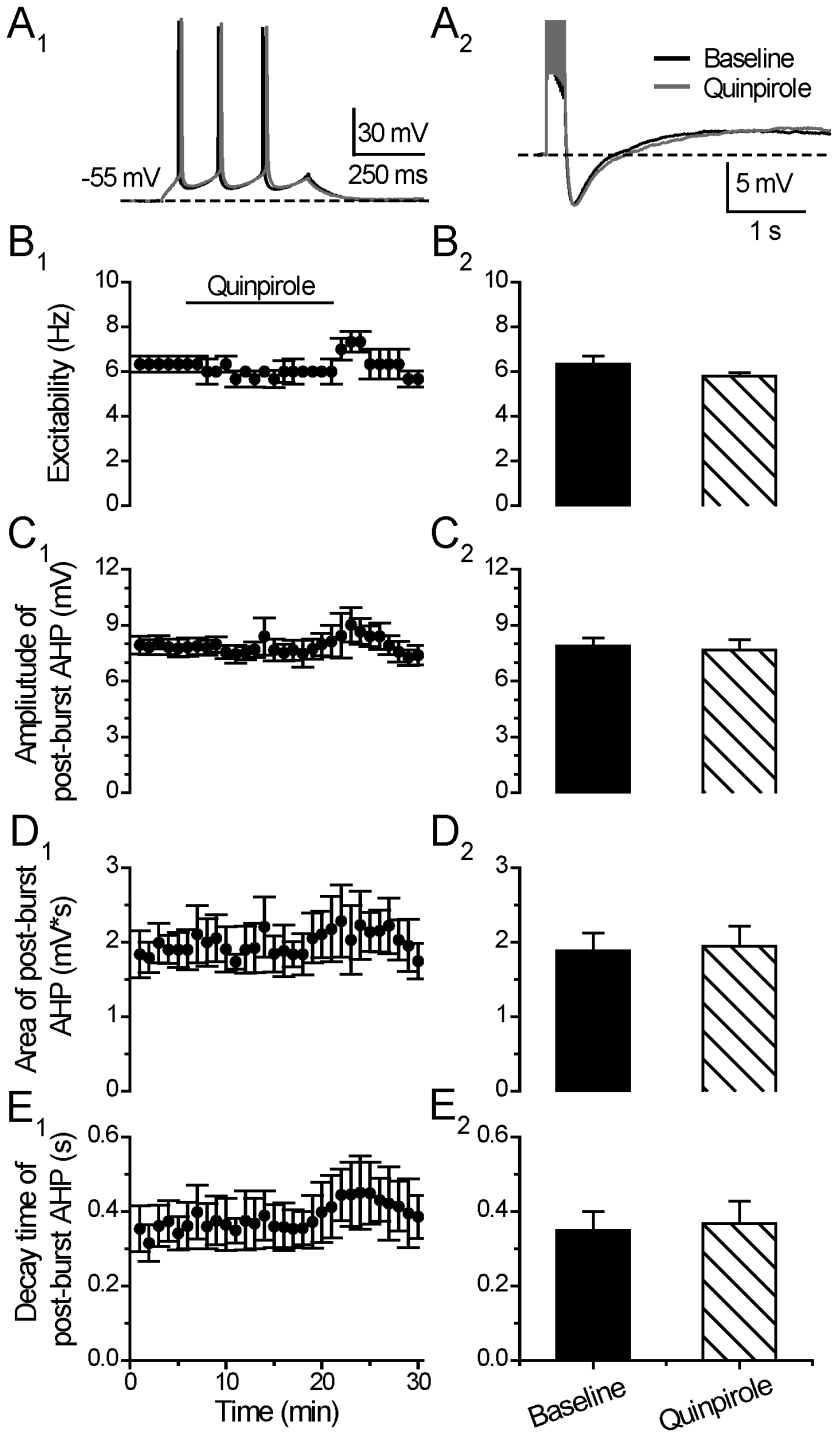
D2/3/4 stimulation does not affect neuronal excitability and post-burst AHP. (**A**) An example of the effect of D2 receptor agonist Quinpirole (10 µM) on neuronal excitability (left) and post-burst AHP (right). The protocols for excitability test and post-burst AHP recording were run alternately every 30 seconds. (**B**) Excitability before, during and after 15-min application of Quinpirole (left). Group histograms showing that Quinpirole does not change the neuronal excitability of mPFC neurons (right) (p>0.05 for Quinpirole vs. baseline, paired *t-*test). (**C**) Amplitude of post-burst AHP before, during and after 15-min application of Quinpirole (left). Group histograms showing that Quinpirole does not change the amplitude of post-burst AHP (right) (p>0.05 for Quinpirole vs. baseline, paired *t-*test). (**D**) Area of post-burst AHP before, during and after 15-min application of Quinpirole (left). Group histograms showing that Quinpirole does not change the area of post-burst AHP (right) (p>0.05 for Quinpirole vs. baseline, paired *t-*test). (**E**) Decay time of post-burst AHP before, during and after 15-min application of Quinpirole (left). Group histograms showing that Quinpirole does not change the decay time of post-burst AHP (right) (p>0.05 for Quinpirole vs. baseline, paired *t-*test).

In the continuous presence of blockers for glutamate and GABA-mediated synaptic transmission, we then investigated the effects of the D2/3/4 receptor agonist quinpirole on neuronal excitability and post-burst AHP in the same tested neurons. As shown in Figure 5, bath-application of quinpirole (10 µM) induced no significant effect on both the neuronal excitability (Excitability: from 6.34±0.36 to 5.80±0.14 Hz, p = 0.16, paired *t* test, n = 6) (Fig. 5A), and the post-burst AHP (Amplitude: from 7.88±0.41 to 7.66±0.55 mV, p = 0.49; Area: from 1.88±0.24 to 1.94±0.27 mV*s, p = 0.60; Decay time constant: 0.35±0.05 to 0.37±0.06, p = 0.32, paired *t* test, n = 6) (Fig. 5B, 5C and 5D), suggesting that D2/3/4 receptors were not involved in the DA regulation on neuronal excitability changes and post-burst AHP. In a few neurons, there appeared to have a “rebound” excitability increase effect after the termination of quinpiorle (5 minutes) but this was not statistically significant (Excitability: 6.80±1.04 Hz, p = 0.33, paired t test, n = 6).

### DA and D1/5-receptor Regulation of Excitability and Post-burst AHP does not Involve HCN

Membrane hyperpolarization induced by post-burst AHP typically lasted >100 ms, which is sufficient to activate I_h_ via hyperpolarization-activated cyclic nucleotide-gated (HCN) channels. Thus, the activation of the Ca^2+^-activated K^+^ current (mediating AHP) overlaps with the activation of the opposing I_h_ current that depolarizes the membrane towards resting potential. The interaction between hyperpolarizing AHP and the depolarizing I_h_ are likely to be responsible for the self-sustained recurrent burst firing in pyramidal cells [31].

#### i) Effect of HCN-channel blocker ZD7288 alone

**Figure 6:**
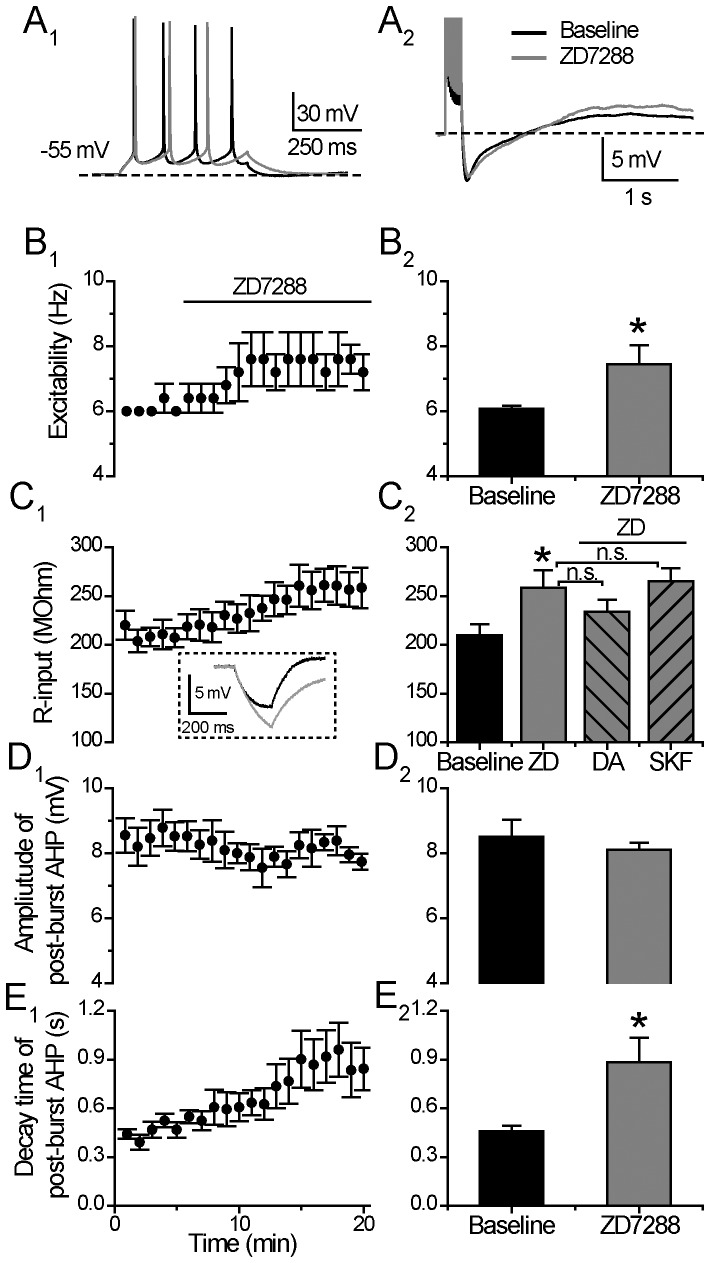
HCN channels are open persistently. (**A**) An example of the effect of HCN channels blocker ZD7288 (20 µM) on neuronal excitability (left) and post-burst AHP (right). The protocols for excitability test and post-burst AHP recording were run alternately every 30 seconds. (**B**) Excitability before and during 15-min application of ZD7288 (left). Group histograms showing that ZD7288 enhances the neuronal excitability of mPFC neurons (right) (*p<0.05 for ZD7288 vs. baseline, paired *t-*test). (**C**) Input resistance before and during 15-min application of ZD7288 (left). In set, the representative voltage responses upon hyperpolarizing current injection (30 pA) in baseline and ZD7288 application. Group histograms showing that ZD7288 enlarges the input resistance significantly (*p<0.05 for ZD7288 vs. baseline, paired *t-*test), DA or SKF 81297 application respectively does not change the input resistance anymore in the presence of ZD7288 (right) (p>0.05 for DA+ZD7288 vs. ZD7288; p>0.05 for SKF 81297+ZD7288 vs. ZD7288, one way ANOVA). (**D**) Amplitude of post-burst AHP before and during 15-min application of ZD7288 (left). Group histograms showing that ZD7288 does not change the amplitude of post-burst AHP (right) (p>0.05 for ZD7288 vs. baseline, paired *t-*test). (**E**) Decay time of post-burst AHP before and during 15-min application of ZD7288 (left). Group histograms showing that ZD7288 prolongs the decay time of post-burst AHP (right) (*p<0.05 for ZD7288 vs. baseline, paired *t-*test).

As shown in Figure 6, when the tested neurons were held at −55 mV, application of ZD7288 (20 µM) alone significantly enhanced the neuronal excitability and increased the input resistance of the examined cells (Excitability: from 6.08±0.09 to 7.44±0.59 Hz, p<0.05; Input resistance: from 209.97±10.03 to 258.46±16.22 MOhm, p<0.05, paired t test, n = 6) (Fig. 6A_1_, 6B and 6C), suggesting that HCN channels were opened for I_h_ current flux at the holding potential (−55 mV), although, at such a more positive holding potential at −55 mV, a portion of the I_h_ current is likely to be deactivated [32]. When HCN channels were fully blocked with ZD7288, the input resistance increased, resulting in a greater membrane voltage deflection upon a given depolarizing pulse and thereby, facilitating neuronal excitability.

Treatment with ZD7288 (20 µM) *alone* significantly prolonged the decay time constant of post-burst AHP (From 0.46±0.04 to 0.88±0.15 s, p<0.05, paired *t* test, n = 6; Fig. 6E), with no effect on the amplitude of post-burst AHP (From 8.50±0.552 to 8.11±0.21 mV, p = 0.16, paired *t* test, n = 6; Fig. 6D), indicating that I_h_ current was activated during the slow and long time course of a post-burst AHP and I_h_ functionally opposed the duration of the AHP.

#### ii) Effects of DA and SKF81297 under HCN blockade

Under a steady-state condition (perfusion of ZD7288 for >10 mins), when HCN channels were blocked by ZD7288 (20 µM) and the cells were held at −55 mV, DA or SKF81297 *alone* did not further change the input resistance (DA/Input resistance: 233.86±11.68 MOhm, p>0.05, one way ANOVA, n = 17; SKF81297/Input resistance: 265.12±13.09 MOhm, p>0.05, one way ANOVA, n = 10; Fig. 6C2).

**Figure 7:**
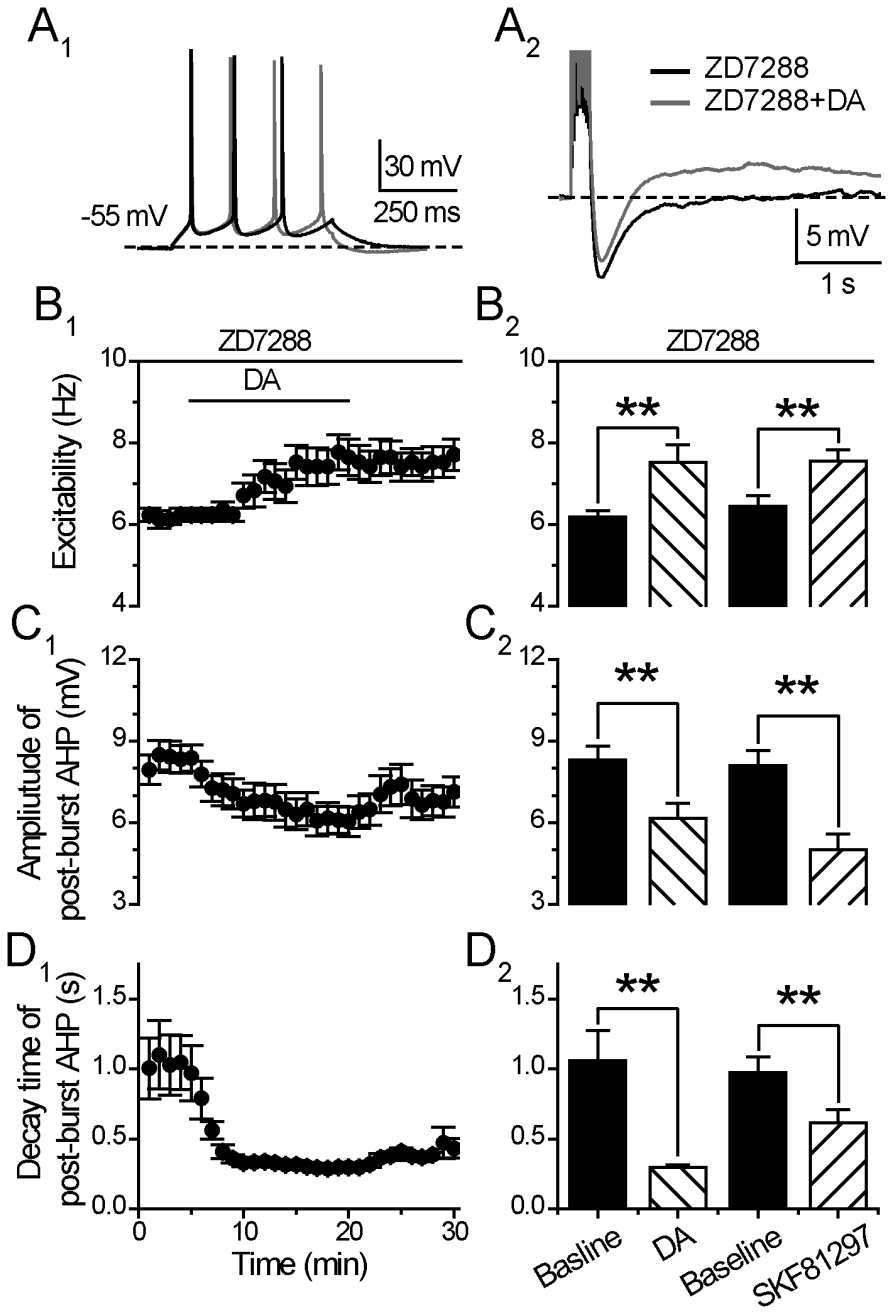
DA and D1/5 regulation of excitability and post-burst AHP does not involve HCN. (**A**) An example of the effect of DA (30 µM) on neuronal excitability (left) and post-burst AHP (right) in the presence of HCN blocker ZD7288 (20 µM). The protocols for excitability test and post-burst AHP recording were run alternately every 30 seconds. (**B**) Excitability before, during and after 15-min application of the DA in the presence of ZD7288 (left). Group histograms showing that DA (30 µM) or D1/5 receptor agonist SKF81297 (10 µM) enhances the neuronal excitability of mPFC neurons in the presence of HCN blocker ZD7288 (right) (**p<0.01 for ZD7288+DA vs. baseline; **p<0.01 for ZD7288+SKF81297 vs. baseline; paired *t-*test). (**C**) Amplitude of post-burst AHP before, during and after 15-min application of the DA in the presence of ZD7288 (left). Group histograms showing that DA (30 µM) or D1/5 receptor agonist SKF81297 (10 µM) reduces the amplitude of post-burst AHP in the presence of ZD7288 (right) (**p<0.01 for ZD7288+DA vs. baseline; **p<0.01 for ZD7288+SKF81297 vs. baseline; paired *t-*test). (**D**) Decay time of post-burst AHP before, during and after 15-min application of the DA in the presence of ZD7288 (left). Group histograms showing that DA (30 µM) or D1/5 receptor agonist SKF81297 (10 µM) reduces the decay time of post-burst AHP in the presence of ZD7288 (right) (**p<0.01 for ZD7288+DA vs. baseline; **p<0.01 for ZD7288+SKF81297 vs. baseline; paired *t-*test).

In the continuous presence of the I_h_ blocker ZD7288 (20 µM), DA (30 µM) still enhanced the neuronal excitability (Excitability: from 6.19±0.15 to 7.53±0.42 Hz, p<0.01, paired *t* test, n = 17; Fig. 7B), and suppressed the post-burst AHP (Amplitude: from 8.31±0.51 to 6.17±0.54 mV, p<0.01; Decay time constant: 4.88±0.75 to 1.54±0.19 s, p<0.01, paired *t* test, n = 17; Fig. 7C and 7D). Further, the DA enhancement of excitability demonstrated no difference in the absence or presence of ZD7288 (Excitability: 119.88±5.01% without ZD7288 vs.121.76±5.75% with ZD7288, p>0.05, unpaired *t* test, n = 14 and n = 17 cells, respectively). Notably, in the presence of ZD7288, the reduction of post-burst AHP amplitude and decay time constant did not recover back to the baseline values after the termination of DA application (Fig. 7D), although these two parameters did recover to baseline in the absence of ZD7288 (see Fig. 2E).

Similarly, under the continuous blockade of HCN channels by ZD7288 (20 µM), the D1/5 receptor agonist SKF81297 (10 µM) also enhanced the neuronal excitability (Excitability: from 6.45±0.25 to 7.55±0.28 Hz, p<0.01. paired t test, n = 10) (Fig. 7B_2_), and suppressed the post-burst AHP (Amplitude: from 8.09±0.57 to 5.02±0.56 mV, p<0.01; Decay time constant: 0.98±0.11 to 0.61±0.10 s, p<0.05, paired t test, n = 10) (Fig. 7C_2_ and 7D_2_). The SKF81297 enhancement of excitability displayed no difference in the absence vs. presence of ZD7288 (Excitability: 114.76±6.07% without ZD7288 vs. 118.70±5.00% with ZD7288, p>0.05, unpaired *t* test, n = 19 and n = 10 cells, respectively).

### DA and D1/5-receptor Regulation of Post-burst Afterdepolarizing Potentials

Spike burst activity not only results in a post-burst AHP, but also afterdepolarizing potential (ADP). Attenuation of AHP has been shown to unveil an afterdepolarization in dopamine neurons and striatal cholinergic interneurons [24], [33]. The interaction of AHP and ADP may overlap in time and are likely to be responsible for the self-sustained recurrent burst firing in pyramidal cells [31].

**Figure 8:**
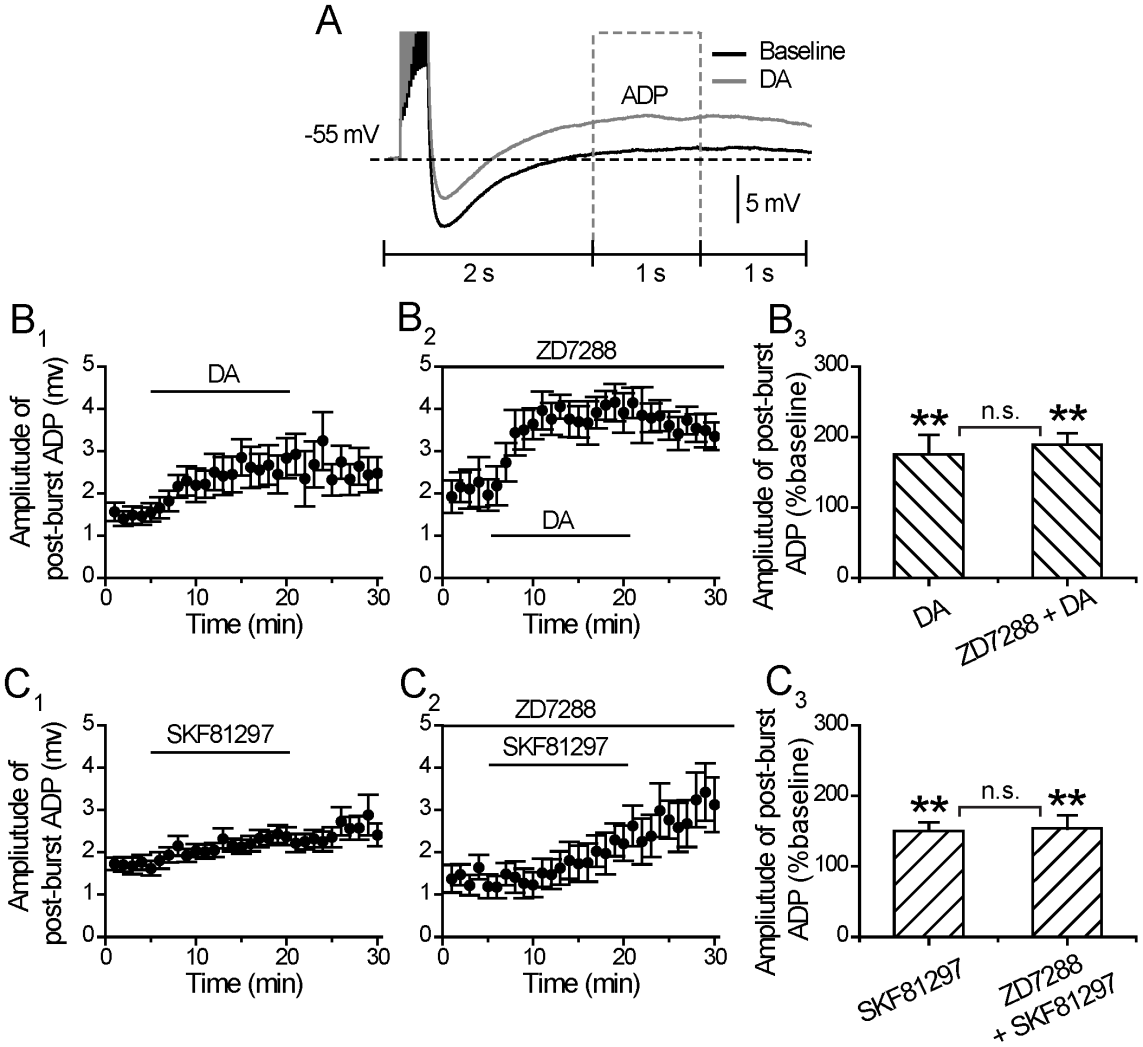
DA and D1/5 regulation of post-burst afterdepolarizing potentials. (**A**) An example of the effect of DA (30 µM) on afterdepolarizing potentials (ADP). (**B**) Amplitude of ADP before, during and after 15-min application of the DA in the absence (left) or in the presence of HCN blocker ZD7288 (20 µM) (middle). Group histograms showing DA enlarges the amplitude of ADP in the absence or in the presence of ZD7288 (right) (**p<0.01 for DA vs. baseline; **p<0.01 for DA+ZD7288 vs. baseline, paired *t-*test) (p>0.05 for DA+ZD7288 vs. DA, one way ANOVA). (**C**) Amplitude of ADP before, during and after 15-min application of SKF81297 in the absence (left) or in the presence of HCN blocker ZD7288 (middle). Group histograms showing that SKF81297 enlarges the amplitude of ADP in the absence or in the presence of HCN blocker ZD7288 (right) (**p<0.01 for SKF81297 vs. baseline; **p<0.01 for SKF81297+ZD7288 vs. baseline, paired *t-*test) (p>0.05 for SKF81297+ZD7288 vs. SKF81297, one way ANOVA).

In this study, DA not only reduced the decay time constant of post-burst AHP, but also augmented the ADP component following the post-burst AHP (Fig. 8A). This may be a direct or an indirect effect of DA, since the emerged enhancement of the ADP could be a result of a DA suppression of the AHP, or DA could directly augment the intrinsic mechanisms that mediate the ADP, but are normally and largely masked by the AHP. In the lateral amygdala, DA has also been shown to induce an ADP after a train of action potential [34].

As mentioned above, in the absence of the HCN blocker ZD7288 (20 µM), we found that application of DA (30 uM) not only resulted in a significant reduction in the decay time constant of the post-burst AHP, but also unveiled an emerging long-duration (>2 s) ADP (ADP Amplitude: from 1.49±0.19 to 2.62±0.43 mV, p<0.01, paired *t* test, n = 14) (Fig. 8B_1_ and 8B_3_). In the presence of ZD7288 (20 µM) the DA augmentation of ADP persisted (Amplitude: from 2.08±0.40 to 3.94±0.33 mV, p<0.01, paired *t* test, n = 17, 14 out of 17 cells showed an increase in ADP) (Fig. 8B_2_ and 8B_3_). No difference was found between DA effect on ADP in the absence and presence of ZD7288 (p>0.05, one way ANOVA, Fig. 8B_3_), suggesting that the DA-induced ADP was also independent of I_h_. Thus, regardless of whether the HCN channels were blocked or not, DA still significantly suppressed the post-burst AHP and enhanced the ADP.

Similar results were obtained when the D1/5 receptor agonist SKF81297 (10 µM) was applied in the absence or presence of ZD7288 (Amplitude of ADP in the absence of ZD7288: from 1.70±0.16 to 2.34±0.18 mV p<0.01, paired *t* test, n = 19; and Amplitude of ADP in the presence of ZD7288: from 1.38±0.23 to 2.05±0.40 mV, p<0.01, paired *t* test, n = 10) (Fig. 8C). No difference was found between SKF81297 effect on ADP in the absence and presence of ZD7288 (p>0.05, one way ANOVA). Notably, during the drug application, the onset of the responses in enhancing the ADP amplitude was faster with DA than the D1/5 agonist.

### DA and D1/5-receptor Regulation of Post-single Spike AHP

**Figure 9:**
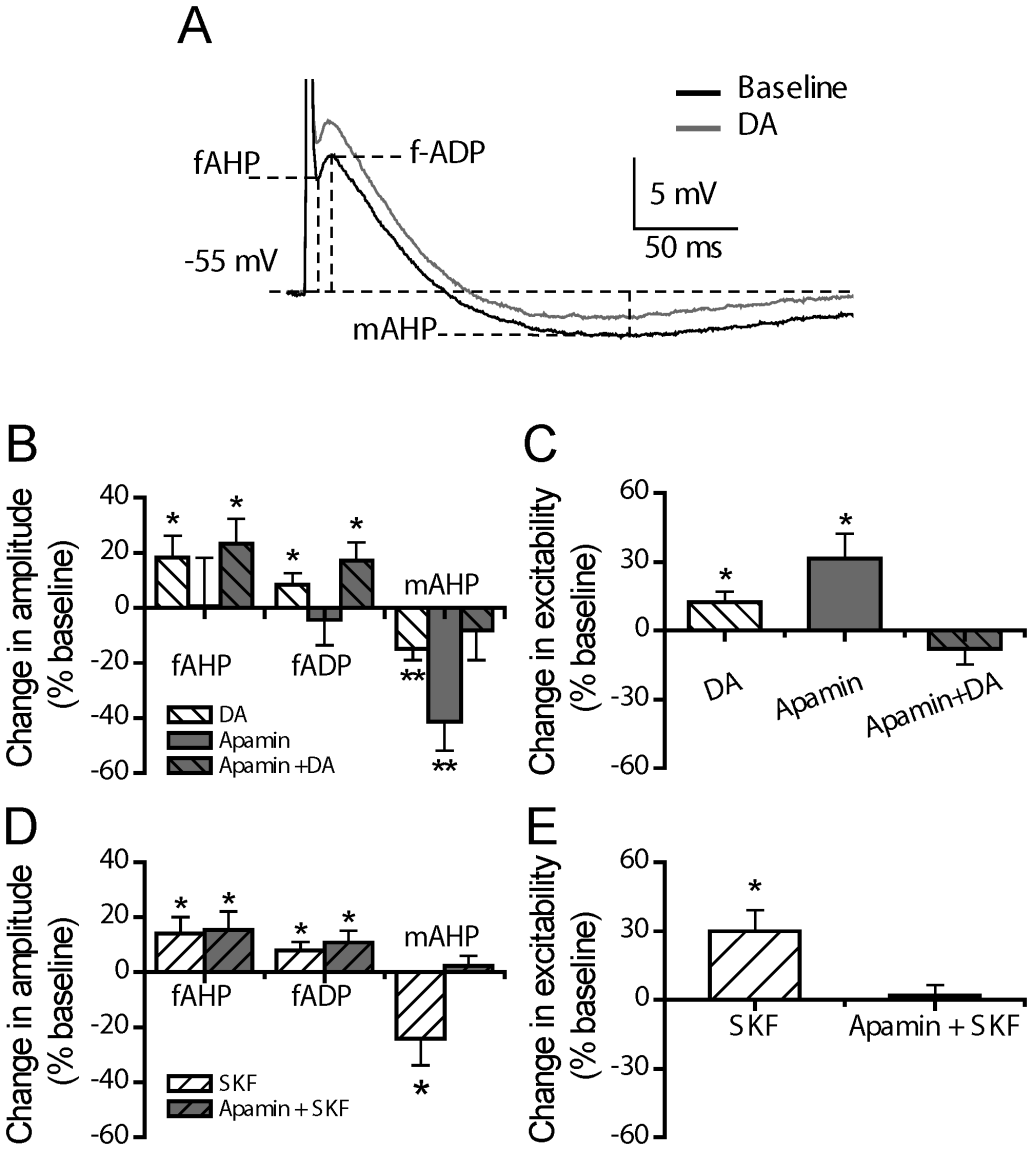
DA and D1/5 regulation of post-spike AHP. (**A**) An example of the effect of DA (30 µM) on the post-spike AHP. (**B**) Group histograms showing that DA enlarges the amplitude of fAHP and fADP, and suppresses mAHP (*p<0.05 for DA vs. baseline, paired *t-*test); apamin (0.1 µM) itself suppresses mAHP (**p<0.01 for apamin vs. baseline, paired *t-*test); in the presence of apamin, DA enlarges the amplitude of fAHP and fADP (*p<0.05 for DA+apamin vs. baseline, paired *t-*test), but does not suppress mAHP anymore (p>0.05 for DA+apamin vs. baseline, paired *t-*test) (For the grey column with back slash, its baseline refers to the data obtained in the presence of apamin before DA application). (**C**) Group histograms showing that DA enhances the neuronal excitability; apamin itself enhances the neuronal excitability (*p<0.05 for DA vs. baseline, paired *t-*test); in the presence of apamin, DA does not change neuronal excitability anymore (p>0.05 for DA+apamin vs. baseline, paired *t-*test) (For the grey column with back slash, its baseline refers to the data obtained in the presence of apamin before DA application). (**D**) Group histograms showing SKF81297 enlarges the amplitude of fAHP and fADP, suppresses mAHP (*p<0.05 for SKF81297 vs. baseline, paired *t-*test); in the presence of apamin, SKF81297 enlarges the amplitude of fAHP and fADP (*p<0.05 for SKF81297+apamin vs. baseline, paired *t-*test), but does not suppress mAHP anymore (p>0.05 for SKF81297+apamin vs. baseline, paired *t-*test) (For the grey column with slash, its baseline refers to the data obtained in the presence of apamin before SKF81297 application). (**E**) Group histograms showing SKF81297 enhances the neuronal excitability; in the presence of apamin, SKF81297 does not change neuronal excitability anymore (p>0.05 for SKF81297+apamin vs. baseline, paired *t-*test) (For the grey column with slash, its baseline refers to the data obtained in the presence of apamin before SKF81297 application).

We have also analyzed the effects of DA and D1/5-receptor activation on post-single spike AHP. As shown in Figure 9A, an evoked single spike in mPFC pyramidal cells is usually followed by a post-spike AHP, which is often obscured by a small but fast post-spike ADP (fADP). According to their temporal appearance, we subdivided the post-single spike events into three components: fAHP, fADP and mAHP.

As shown in Figure 9B, compared to baseline recording, DA application *alone* (30 µM) resulted in an enhancement of both fAHP and fADP (ΔfAHP: 18.09±8.17%, p<0.05; ΔfADP: 8.59±3.89%, p<0.05, paired *t* test, n = 12), and a suppression of mAHP (ΔmAHP: −14.94±4.07%, p<0.01, paired *t* test, n = 12).

On the other hand, apamin *alone* (0.1 µM) did not change fAHP and fADP (ΔfAHP: 0.41±17.83%, p>0.05; ΔfADP: −4.04±9.25%, p>0.05, paired *t* test, n = 6), but suppressed the mAHP (ΔmAHP: −41.22±10.48%, p<0.01, paired *t* test, n = 6). Thus, mAHP, but not fAHP and fADP, was apamin-sensitive.

In the continuous presence of apamin (0.1µM), application of DA still enhanced fAHP and fADP (ΔfAHP: 23.26±9.17%, p<0.05; ΔfADP: 17.07±6.87%, p<0.05, paired *t* test, n = 6), but did not change the mAHP (ΔmAHP: −8.30±10.71%, p>0.05, paired *t* test, n = 6), during the last 5 min of 15 min DA application. This result suggests that DA suppressed an apamin-sensitive mAHP.

The DA effects on the fAHP, fADP and mAHP components were mimicked by D1/5-receptor stimulation. As shown in Figure 9D, the D1/5-receptor agonist SKF81297 *alone* (10 µM) resulted in an enhancement of fAHP and fADP (ΔfAHP: 14.16±5.96%, p<0.05; ΔfADP: 8.76±3.00%, p<0.05, paired *t* test, n = 6) and a strong reduction of mAHP (ΔmAHP: −24.22±9.59%, p<0.05. paired *t* test, n = 6). In the continuous presence of apamin (0.1 µM), SKF81297 enhancement of fAHP and fADP still occurred (ΔfAHP: 15.44±6.63%, p<0.05; ΔfADP: 10.78±4.38%, p<0.05, paired t test, n = 7), but its reduction of mAHP no longer occurred (ΔmAHP: 2.36±3.42%, p>0.05, paired t test, n = 7). Again, this data further suggest that DA, via D1/5 receptor activation suppresses an apamin-sensitive post-spike mAHP.

We next analyzed the effects of DA and D1/5-receptor stimulation on alternatively tested excitability and post-single spike AHP (these cells have never been tested for post-burst AHP). As shown in Figure 9C, DA *alone* (30 µM) enhanced the neuronal excitability (from 6.80±0.54 to 7.67±0.60 Hz, 12.37±4.45%, p<0.05, paired t test, n = 12); and apamin *alone* (0.1 µM) also produced an increase in excitability (from 7.36±0.82 to 9.60±1.17 Hz, 31.27±11.00%, p<0.05, paired t test, n = 6). However, in the continuous presence of apamin (0.1 µM) that blocked the post-spike mAHP, DA no longer altered the neuronal excitability (from 7.92±0.71 to 7.20±0.55 Hz, −7.95±6.79%, p>0.05, paired *t* test, n = 6). Apamin occluded the effects of DA on neuronal excitability.

Similarly, while SKF81297 *alone* (10 µM) enhanced the neuronal excitability (from 6.00±0.54 to 7.80±0.49 Hz, 30.00±8.99%, p<0.05, paired *t* test, n = 6). Such enhancement no longer appeared in the continuous presence of apamin (0.1 µM) (from 6.97±0.69 to 7.09±0.53 Hz, 1.80±5.43%, p>0.05, paired *t* test, n = 7) (Fig. 9E). In other words, apamin blockade of AHP occluded the effects of DA and SKF to enhance neuronal excitability. Thus, these results indicate that DA enhancement of neuronal excitability is mainly mediated by apamin-sensitive post-spike mAHP current.

## Discussion

**Figure 10:**
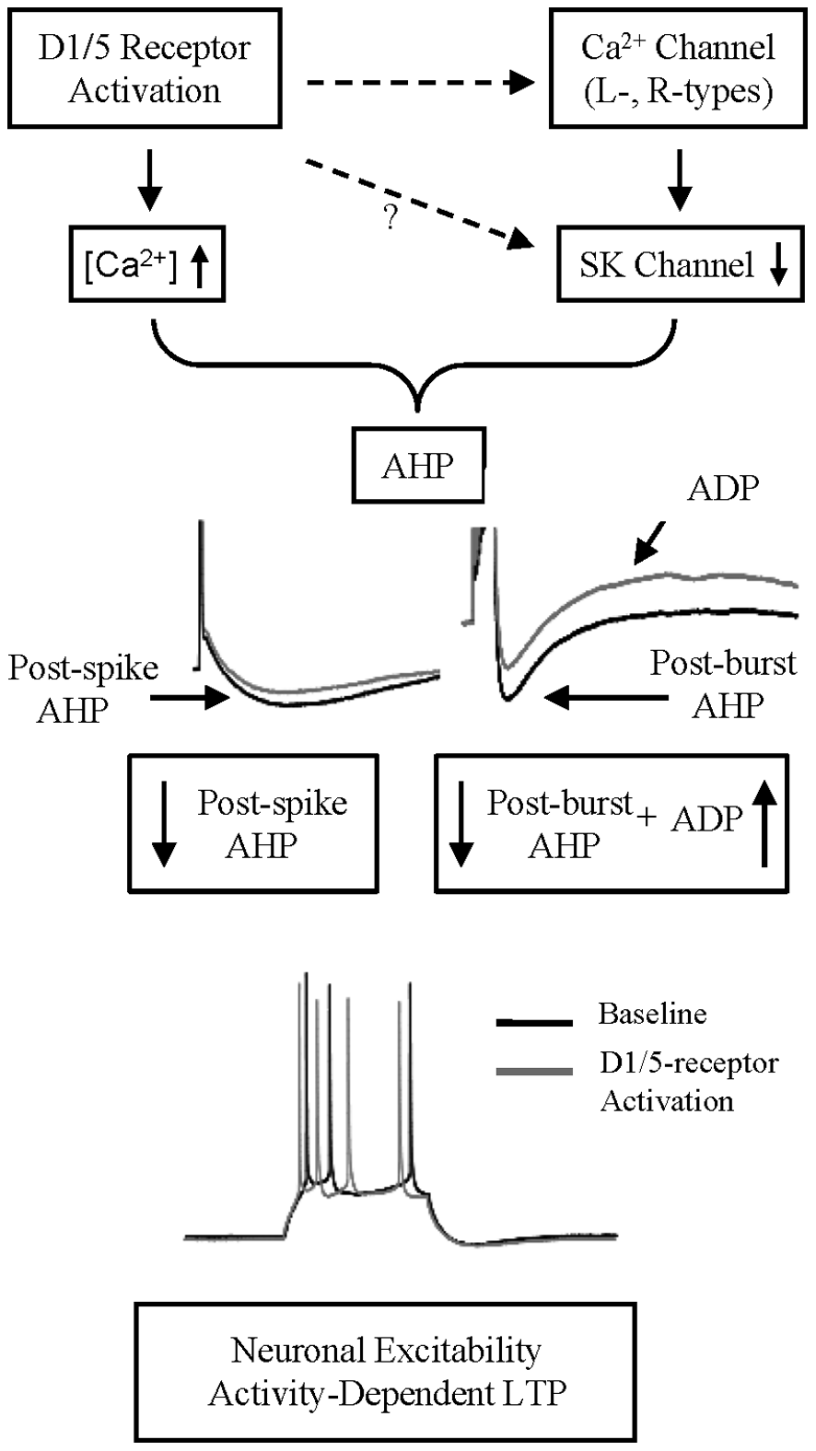
Model of D1/5 receptor-mediated increased neuronal excitability. Activation of D1/5 receptor depresses post-spike mAHP and post-burst AHP, and augments ADP, contribute to the facilitation of intrinsic neuronal excitability in mPFC cells.

The present study first replicated previous findings to show that DA enhances the neuronal excitability of pyramidal cells of prepubertal rat PFC during and after its application, and such enhancement was mimicked by activation of D1/5, but *not* D2/3/4, receptors. The DA-enhancement of neuronal excitability was accompanied by a suppression of the apamin-sensitive post-burst AHP, and the post-spike mAHP to reduce spike frequency adaptation. The additional enhancement by DA via D1/5 receptor activation of a post-AHP afterdepolarization (ADP) also contributes to the overall enhancement of neuronal excitability in PFC neurons (Fig. 10).

### Methodological Considerations

There are a few notable differences between our approaches in the present study, when compared with previously published studies on the influence of DA on neuronal excitability in PFC neurons. The overall magnitude of the excitability changes reported here is smaller than that reported in the previous studies [4], [29]. This was attributed to the time points where the responses were taken. In the present study, the changes in neuronal excitability were measured during the last 5 min of the 15-min drug application period, when the typical maximal DA/D1 effect has not been reached, whereas in the previous study [4], the total mean changes in neuronal excitability were determined during the entire period of drug application and including the 30-min post-drug period, when the maximal effect of DA/D1 effects were achieved. In the present study, we chose the last 5 min of DA/D1 agonist application to quantify neuronal excitability change so that we could compare it with the peak changes in post-burst AHP in the same time window. Our data showed that D1/5 suppression of post-burst AHP contributed to the increase neuronal excitability in the majority of the PFC neurons as analyzed during this 5 min period.

In addition, since the afterhyperpolarization is temperature-sensitive and can be evoked maximally at lower (eg, room) temperature [35], [36], we adopted the approach of whole-cell recordings at room temperature of 25°C and current-clamped the neurons at −55 mV in order to maximize simultaneously the activation of AHP and neuronal excitability. In contrast, Chen et al. [4] carried out whole-cell recordings at a mean bath temperature of 33°C and current-clamped the neurons constantly at −65 mV [4]. Under both temperatures (25 or 33°C) or holding potentials, the profile of the increase in neuronal excitability by DA or its D1/5 agonist did not differ.

### DA or D1/5-receptor Activation Enhances Neuronal Excitability

DA has been reported to exert conflicting influences on neuronal excitability in neurons from different, and even in the same brain region(s). A few previous studies have reported that DA negatively regulates neuronal excitability in hippocampus, entorhinal cortex and striatum [37]–[39] while enhances neuronal excitability in the hippocampus [40]. In the rat PFC, DA enhances the overall neuronal excitability in a prolonged manner (beyond the time when the compound application was terminated) [4], [29], [30], [41], [42], and suppresses intracellularly-evoked simulated EPSPs [43](also see Table 1 for a summary).

Not all the recorded cells in our study exhibited excitability enhancement upon DA or D1/5 agonist application. This suggests that there might be two separate DA responsive and non-responsive pyramidal cells. We did not find their correlation with the amplitude of post-burst AHP or postnatal age (data not shown). A recent study in BAC (bacteria artificial chromosome) transgenic mice suggest that in layer V pyramidal D1+ neurons that fire in spike bursts upon intracellular depolarization pulse activation, has higher input resistance and with smaller Ih than D1- PFC neurons, and shows an increased neuronal excitability in response to a D1 agonist stimulation [30]. In the present analyses, the cluster of neurons that showed increased excitability by D1 agonist SKF81297 also showed the electrophysiological features of typical D1+ neurons, i.e. higher input resistance (p = 0.023) and smaller Ih (p = 0.052) compared with the non-responding cells.

The excitability of some of the cells were partially enhanced during the application of DA or its agonist, but the peak increase excitability occurred after the termination of DA or its D1/5 agonist, when the initially suppressed AHP (during DA or D1/5 agonist application) has already returned back to control levels. This suggested that the suppressed AHP may be contributing to the early phase of increase excitability, but the post-DA excitability increase and maintenance may be mediated via other mechanisms.

### DA or D1/5-receptor Activation Suppresses Post-burst Afterhyperpolarization

Both DA and D1/5 agonist were shown to suppress post-burst AHP in the present study. The D1/5 receptor antagonist SCH23390 blocked SKF81297 suppression of post-burst AHP, suggesting SKF81297 specifically acts on D1/5 receptors to inhibit post-burst AHP. Although, D2/3/4 receptor activation led to no change in the neuronal excitability of prepubertal (postnatal days ≤35 days) PFC pyramidal (present study) and interneurons [44], considering that D2/3/4 receptor modulation of PFC function can be age-dependent [45], our present conclusions for an absence of D2/3/4 effects in neuronal excitability may be restricted to prepubertal PFC neurons. Nevertheless, the D1/5 receptor mechanisms in enhancing neuronal excitability appeared to be less dependent on the age of rat PFC pyramidal neurons (see Table 1). A recent study also showed that norepinephrine induced long duration synaptic depression in PFC is similar in young and adult rats [46].

D1/5 receptor activation-induced increase of neuronal excitability can be through multiple cellular pathways in both PFC pyramidal cells and interneurons, including modulation of K^+^, Ca^2+^ and Na^+^ channels [41], [47]–[50]. There are additional interactions between D1 and NMDA receptors [51], [52], PKA-dependant modulation of K^+^ channels [29], [47] and intracellular Ca^2+^ dependent signaling [4], [26] that mediate the D1-dependent increase in neuronal excitability. In addition to all these mechanisms, we found that D1/5 receptor activation suppressed the Ca^2+^ activated K^+^ current that mediates post-spike/burst AHP, to result in an increase in neuronal excitability.

### Functional Roles of Intracellular Ca^2+^ in Mediating D1/5 Receptor Mediated Changes in AHP and Neuronal Excitability

The enhanced excitability by DA or D1/5 receptor agonist is dependent on intracellular Ca^2+^ elevation in the PFC [4]. Yet, both Ca^2+^ influx and ryanodine receptor-mediated Ca^2+^ release from intracellular Ca^2+^ stores are known to activate AHP [53], which will result in a *decreased* excitability. Hence, the time course of this D1/5 receptor mediated intracellular Ca^2+^ changes, the physical compartmentalization of the intracellular pool(s) of Ca^2+^ [54] that led to SK channels activation, as well as the dynamic sensitivity of the SK channel to the Ca^2+^, may all contribute differentially to the complex mechanisms that mediate intracellular Ca^2+^ dependent neuronal excitability changes in PFC neurons through AHP modulation. Furthermore, we cannot rule out the possibility that D1/5 receptor activation may also suppress SK channels directly, but independent on the early intracellular Ca^2+^ level rise following the D1/5 receptor activation. An understanding of the time course and dynamic relationships of DA-dependent Ca^2+^ elevation that leads to SK channel activation, DA inhibition of SK channel (and ADP activation, see below), would provide further insight into a fuller understanding of how DA increases iCa^2+^ and suppress a Ca^2+^ dependent SK channel to enhance neuronal excitability.

### The Roles of I_h_ and ADP in DA Enhancement of Neuronal Excitability

Membrane hyperpolarization from the post-burst AHP can activate HCN channels that mediate I_h_. The depolarization mediated by I_h_ may augment an ADP, especially following DA D1/5 receptor activation. As the voltage activation of these three currents overlaps, the resulting post-burst AHP is a product of an interaction among Ca^2+^- and apamin-sensitive AHP currents, I_h_ and ADP.

HCN channel has a critical role in normalizing the distal dendritic EPSPs with respect to somatic EPSPs, and Ca^2+^ spikes [55]–[57]. DA is known to modulate HCN channels to *suppress* neuronal excitability in layer I interneurons of the PFC and in the pyramidal neurons of rat entorhinal cortex [37], [58]. In our present study, after blocking the HCN channels with ZD7288, we showed that DA or D1/5-receptor activation still clearly suppressed the post-burst AHP, and unmasked an ADP. It is known that HCN channels in the cortical pyramidal dendrites show a gradient distribution, with the densest distribution along apical dendrites in the superficial layers [59], [60], where D1 receptors are co-located [61]. It is possible that DA modulation of HCN channels is brain-region and cortical-layer specific. Although our data suggests that the increased neuronal excitability is independent of I_h_ in Layer V rat PFC pyramidal neurons, the measurement of neuronal excitability using somatic whole-cell recording by somatic current injection could not fully evaluate the exact roles of the DA on HCN channels in neuronal excitability changes in PFC pyramidal neurons because of the poor voltage control due to ‘space-clamp’ problem.

DA and D1/5 receptor activation also unveiled a post-AHP ADP. Activation from sub-threshold voltage of this slow ADP could normally be prevented by the post-burst AHP and this ADP could be unveiled when the AHP is suppressed. The induction of ADP is triggered by Ca^2+^ influx in the neocortex [62], [63], by a decrease of K^+^ channel activity specifically in the olfactory cortex or lateral amygdala [34], [64], or by an increase in a persistent Na^+^ current in hippocampal CA1 and striatal interneurons [24], [65]. The activated voltage and duration of the unveiled ADP in the present study resembled a dendritic ‘hump’ potential generated in the proximal dendrites [66]. D1/5 receptor stimulation, via PKA activation, has been shown to potentiate this depolarizing ‘hump’ potential, which is hypothesized to provide a mechanism to boost sub-threshold EPSPs to fire spike burst [50]. In a single-channel study in pyramidal PFC neurons, it was shown that a D1/5 agonist increased the open probability for a sub-threshold persistent Na^+^ current, which can contribute to this ADP [41], [67], [68]. Functionally, ADP has been proposed to have a role in short-term memory storage [69] and the DA-induced ADP in rat lateral amygdala neurons plays a crucial role in fear conditioning [34].

### Functional Roles of DA Modulation of Neuronal Excitability

In the Layer V pyramidal PFC neurons, following a spike train, several currents, including AHP, I_h_ and ADP, are activated. Together, these currents play a concerted role in regulating the generation of the next spikes to fire and thereby, help to encode spike codes in the PFC. The DA enhancement of neuronal excitability via D1/5-receptor activation does not involve HCN channels (I_h_). Instead, DA can suppress Ca^2+^ influx via inhibition of dendritic L- or N-type Ca^2+^ channel [50], [70] that normally would activate SK channels to mediate the post-burst AHP [71], [72]. In addition, DA, via D1/5 receptor also increase intracellular Ca^2+^ [4], [34], [50] or a persistent Na^+^ current [68], [73], which can contribute to the afterdepolarization (ADP) following the AHP [50]. Since DA release in PFC is known to be associated with working memory and rule learning [74], [75], the DA suppression of post-burst AHP, along with an augmentation of an ADP, to promote spike firing to result in a long-term potentiation of neuronal excitability, may represent a DA-dependent rule-based learning, which subsequently is being translated to action outcomes in the PFC [75]–[77]. It appears that the prepubertal PFC is endowed with such functional mechanisms that can be fully engaged for the increasing higher cognitive demands during the process of maturation.
